# Exploring the role of the electrically evoked Vestibulo-Ocular reflex in vestibular implant surgery

**DOI:** 10.1007/s00405-025-09635-y

**Published:** 2025-08-13

**Authors:** Stan C.J. van Boxel, Bernd L. Vermorken, Benjamin Volpe, Nils Guinand, Angélica Perez-Fornos, Elke M.J. Devocht, Raymond van de Berg

**Affiliations:** 1 Department of Otorhinolaryngology and Head and Neck Surgery, Division of Vestibular Disorders, Maastricht University Medical Center, Maastricht, The Netherlands; 2 Mental Health and Neuroscience Research Institute (MHeNs), Maastricht University, Maastricht, The Netherlands; 3 Service of Otorhinolaryngology Head and Neck Surgery, Department of Clinical Neurosciences, Geneva University Hospitals, Geneva, Switzerland

## Abstract

**Purpose:**

Vestibular implantation holds considerable potential as a therapeutic strategy for individuals with vestibular loss. A correct position of the stimulation electrodes is essential for effective implant functionality. For vestibular implants targeting the semi-circular canals, Vestibulo-Ocular Reflex testing (VOR testing) is used to evaluate functionality postoperatively. It might also be an effective tool for intraoperative decision making related to electrode placement. This study aimed to evaluate the predictive value of intraoperative VOR testing for the postoperative vestibular implant response. This would be the first step in exploring the usability of the VOR as intraoperative electrode placement indicator.

**Methods:**

Nine patients with bilateral vestibulopathy were implanted with an investigational vestibulo-cochlear implant. Vestibulo-ocular reflexes (VOR) were electrically evoked using the implant, both intra- and postoperatively, and recorded using video-oculography. The correspondence between the intra- and postoperative measurements was evaluated. This included the presence of the VOR, activation threshold, eye velocity and alignment. Furthermore, the correlation between the intraoperative peak eye velocity and stimulation amplitude was assessed.

**Results:**

It was found that the presence of an intraoperative VOR has a high positive predictive value (1.0) for predicting the presence of a postoperative VOR. Additionally, the intraoperative VOR showed a high, though not perfect, negative predictive value (0.86) for predicting the absence of a postoperative VOR. The VOR activation threshold was higher, eye velocity was lower, and alignment differed on individual level, in the intra-operative measurements compared to the post-operative measurements. The intraoperative VOR peak eye velocity was related to stimulation amplitude.

**Conclusion:**

Intraoperative VOR responses during vestibular implant surgery differ from postoperative responses, likely due to general anaesthesia. Despite this discrepancy, the presence of an intraoperative VOR is a strong predictor of postoperative VOR presence. Furthermore, intraoperative VOR eye velocity increases with higher stimulation amplitudes. These findings suggest that intraoperative VOR testing may aid in optimizing electrode placement during vestibular implant surgery.

**Supplementary Information:**

The online version contains supplementary material available at 10.1007/s00405-025-09635-y.

## Introduction

The vestibular implant, a novel treatment approach for patients with loss of vestibular function, is studied by multiple research groups. This neuroprosthesis follows a similar concept to that of the cochlear implant [1]. The most widely used investigational vestibular implant concept detects head movements, and provides vestibular information by modulating electrical currents applied to the nerve endings in the ampullae of the semi-circular canals [2–6]. Stimulation is provided with the intention of restoring critical vestibular functions, such as gaze stabilization by activating the vestibulo-ocular reflex (VOR) [3].

Positioning the electrodes close to the target ampullary nerve ends seems essential to enable effective stimulation (e.g. stimulating the VOR) [7, 8]. Furthermore, an optimal electrode position is expected to facilitate increased selectivity of the stimulation, by limiting undesired current spread to neighboring nerve ends (facial and adjacent ampullary nerves) [9].

However, correctly positioning the electrodes during vestibular implant surgery, is challenging. The insertion is (partially) blind, since the electrode is inserted through a small fenestration in the semicircular canal. As a result, the precise electrode position in the semicircular canal remains uncertain without the use of additional methods [7]. The electrode position can be verified by several methods. Pre- and intraoperative imaging were used to optimize electrode placement [10]. However, this approach can be costly and time-consuming. Moreover, prolonged opening of the canal fenestration might potentially affect residual hearing [7, 11]. Telemetry (specifically ECAP recording) could also be considered. Unfortunately, its predictive value for the postoperative response is limited [12]. As an alternative, the VOR response might be used to evaluate vestibular electrode position.

Measuring the VOR, using video-oculography, is a common way of evaluating vestibular function and the response to vestibular stimulation. During vestibular implant surgery, the electrode can already stimulate the vestibular nerve, which could activate the VOR. It could be hypothesized that this facilitates optimal electrode positioning. For example, during surgery the electrode can be stimulated at different positions in the semicircular canal. The position with the desired VOR characteristics (see below), might be the ‘optimal’ electrode position. Four key aspects of the VOR offer potential applications for intraoperative electrode verification, namely its presence, its threshold, its eye velocity and its (mis)alignment. These aspects will now be discussed.

Firstly, if the VOR can be evoked, its presence indicates that the electrode is sufficiently close to the ampullary nerve to trigger a response. Nevertheless, the mere presence of a response does not necessarily imply optimal positioning. Secondly, the current threshold required for VOR activation may serve as a more precise indicator of the electrode’s proximity to the ampullary nerve. A lower activation threshold suggests closer proximity, as less charge is needed to elicit a response. Thirdly, the eye velocity of the VOR serves as a measure of stimulation effectiveness. This is based on the observation that the postoperative eye response velocity correlates with stimulation intensity [9]. A larger distance between electrode and target neurons potentially reduces stimulation effectiveness. In consequence, when an electrode is activated at a constant supra-threshold stimulation intensity during intraoperative manipulation, a higher eye velocity may indicate a more optimal electrode position. Lastly, the alignment of the VOR can potentially be used to verify whether the electrode position is optimal for the targeted nerve end. The VOR should ideally align with the orientation of the stimulated semicircular canal (Ewald’s first law). Unintentionally activating adjacent ampullary nerve(s) can cause misalignment in the response, making it important to consider both the eye velocity and the (mis)alignment. For instance, an electrode positioned close to a neighboring nerve can produce a strong eye response with a low threshold, yet its position is not optimal.

It should be noted that the presence and characteristics of the VOR are affected by general anesthesia [13, 14]. This compromises the value of using VOR measurements intraoperatively. Therefore, this study aimed to evaluate the predictive value of intraoperative VOR measurements for the postoperative functionality of the vestibular implant. This could in turn be predictive for electrode position. Four VOR characteristics were evaluated. Firstly, it was hypothesized that the intraoperative VOR offers predictive value for the presence of a postoperative VOR. Similarly, intra- and postoperative activation thresholds, eye velocity and alignment were compared to evaluate their predictive value. Additionally, the correlation between the intraoperative eye velocity and the electrical stimulation level was assessed. If the intraoperative VOR would reliably reflect the postoperative functionality, this would imply that the intraoperative VOR could be used for intraoperative electrode positioning.

## Methods

### Subject and implant characteristics

This study was conducted as part of the VertiGo!-trial (ClinicalTrials.gov Identifier: NCT04918745). The protocol describing subject inclusion, surgery and implant was published by Vermorken et al. [15]. Until 2023, nine bilateral vestibulopathy patients with severe sensorineural hearing loss, in at least the ear to be implanted, received an investigational vestibulo-cochlear implant (supplied by MED-EL, Innsbruck, Austria). Subject characteristics are summarized in Table 1. The implant consists of three vestibular electrode leads (each with one electrode contact), inserted in the ampulla of each semicircular canal, and a cochlear electrode lead inserted in the cochlea (containing nine electrode contacts). Vestibular target nerves were the lateral ampullary nerve (LAN), superior ampullary nerve (SAN) and posterior ampullary nerve (PAN).

**Table 1:** Subject characteristics

Subject ID	Sex	Age at implantation (years)	Etiology bilateral vestibulopathy	Duration of symptoms (years)	Year of implantation	Implanted side
VCI-1	Female	54	DFNA-9	7	2021	R
VCI-2	Male	65	Auto-immune (CREST)	21	2021	R
VCI-3	Male	52	DFNA-9	30	2022	L
VCI-4	Male	66	DFNA-9	10	2022	R
VCI-5	Male	28	Idiopathic	4	2022	R
VCI-6	Male	66	M. Meniere	25	2022	R
VCI-7	Female	62	DFNA-9	6	2022	L
VCI-8	Male	63	Skull base fracture	< 1	2023	R
VCI-9	Female	62	Skull base fracture	< 1	2023	R

### Surgery and experimental setup

Vestibular electrode implantation was performed using the intralabyrinthine approach [15]. Real-time fluoroscopy-guidance and pre- and intraoperative 3D images (i.e., CT) were used to optimize and verify electrode placement within 1.5 mm of the ampulla [10]. Propofol 6 mg/kg/h and Remifentanil 0.35 mcg/kg/min were administered during the surgery to maintain anesthesia. Before the start of the VOR experiment, Propofol was stopped, and Remifentanil was continued at the same dosage.

Vestibulo-ocular reflexes (VOR) were electrically evoked using the implant, both intra- and postoperatively, and recorded using video-oculography. Intraoperative experiments were conducted at the end of the surgery, following the completion of electrode insertion and fixation. Postoperative measurements were conducted in single sessions between one and three months after surgery.

### Vestibular stimulation

The implant was controlled using the associated interface and the clinical cochlear implant software (MAX-box and Maestro, MED-EL, Innsbruck, Austria). Stimulation signals were transmitted through a radiofrequency coil placed on the implant. Vestibular electrode stimulation consisted of two second pulse trains of eight, symmetric, biphasic, rectangular, cathodic first pulses. Pulses had a phase duration of 200 µs and an interphase gap of 2.1 µs. Stimulation pulses were delivered at a rate of 400 pulses per second.

Presence of a VOR was determined by visual inspection of the eye recordings, in consensus by at least three of the authors. For intraoperative measurements, stimulation intensity started at 1000 cu (current unit, with 1 cu ~ 1 mA), which was also the maximum stimulation level. Activation thresholds were determined by iteratively decreasing stimulation in steps of 100 cu in case a VOR was observed. The stimulation was increased with 50 cu in case the VOR was no longer observed, until the VOR was present again. Due to time constraints during surgery, the full range between 1000 cu and threshold was not always assessed. During postoperative measurements, stimulation was started at 50 cu. Stimulation was increased in steps of 50 cu (to avoid patient discomfort), when no VOR was observed, and decreased by 25 cu in case of a VOR response. Threshold amplitudes were tested twice in both the intra- and postoperative sessions. Postoperatively, after establishing threshold level, stimulation amplitude was stepwise increased (by 50 cu), to find the upper comfortable limit. The upper comfortable limit was defined as the highest stimulation level without patient discomfort or facial nerve stimulation.

### Video-oculography

To record the characteristics of the VOR in response to vestibular stimulation, vestibulo-oculography was performed using VideoFrenzel goggles (VisualEyes™, Interacoustics, Middelfart, Denmark). The goggles prevented visual fixation during the postoperative session.

For the intraoperative measurements, the eyes were kept open using eye spacers. Patient participation is needed for the eye tracking calibration procedure, which was intraoperatively not feasible due to the general anesthesia. Therefore, no exact peak eye velocities, in degrees per second, could be obtained intraoperatively. As an alternative, the velocity was calculated in pixels per second. This approach enabled comparisons within sessions (goggles in same position), but not across sessions (due to variations in goggle positioning). Calibration was possible postoperatively, facilitating peak eye velocity assessment. The peak eye velocity was defined as the maximum velocity, in the direction of the movement, of the slow phase of the nystagmus. Anesthesia suppressed the fast phases of the nystagmus, resulting in only a single slow phase. Therefore, the first nystagmus beat was used to facilitate a valid comparison between intra- and postoperative measurements.

The alignment was determined with a range from 0 to 360 degrees, from both intra- and postoperative recordings. In this range, 0 degrees was defined as a purely horizontal movement to the left (from the patient’s perspective), and 90 degrees a purely vertical upwards movement.

### Data analysis

Firstly, intra- and postoperative VOR presence was compared, initially using a contingency table and McNemar’s test. Furthermore, the predictive value of the intraoperative VOR for the presence or absence of a postoperative VOR was assessed by calculating the positive and negative predictive values. The positive predictive value was calculated by dividing the number of electrodes with an intraoperative VOR that also demonstrated a postoperative VOR by the total number of cases with an intraoperative VOR response. The positive predictive value ranges from zero to one, with a value of one indicating that if an intraoperative VOR is detected, there is a 100% chance of also detecting a postoperative VOR. The negative predictive value was calculated by dividing the number of cases without a VOR response, both intra- and postoperatively, by the total number of cases that did not show an intraoperative VOR response. A negative predictive value close to one suggests that if no VOR response is detected intraoperatively, the absence of a postoperative VOR response is highly likely.

Secondly, intra- and postoperative activation thresholds were compared to evaluate their reliability as an intraoperative response measure. Differences in activation thresholds intra- and postoperatively were tested for normality using the Shapiro-Wilk test and evaluated using a paired two-sided t-test.

Thirdly, it was assessed whether the stimulation amplitude influenced intraoperative eye velocity by comparing peak eye velocities across a range of stimulation levels. Additionally, the intraoperative and postoperative VOR eye velocities were compared. For each electrode, the eye velocity of the postoperative session was subtracted by the intraoperative eye velocity, both at the postoperative upper comfortable limit amplitude. Since intraoperative calibration was not possible, eye velocities were compared in terms of pixels per second. Due to variations in the positioning of the goggles between the intra- and postoperative sessions, a strict quantitative comparison was not feasible. However, the correlation between velocity in pixels per second and degrees per second was evaluated (on postoperative data, for which both were available). A strong correlation suggested that any differences observed in eye velocities between the intra- and postoperative sessions were likely to reflect true velocity differences.

Fourthly, VOR alignment was evaluated by pairwise comparison between the intra- and postoperative measurements at equal stimulation amplitudes (the postoperative upper comfortable limit). Additionally, a comparison was made at threshold level plus 100 cu. As the general anesthesia affects the response, a stimulation level relative to the threshold provided a comparison that approximated equivalent effectiveness of stimulation. Differences were tested for normality using the Shapiro-Wilk test and evaluated using a paired two-sided t-test. For simplicity purposes, and since torsional components could not be obtained in the current setup, desired alignment was 0° for LAN (for right implantation, 180° for left), 90° for SAN and 270° for PAN. Misalignment was defined as the difference between the direction of the VOR and the desired orientation.

### Ethical considerations

The VertiGO!-trial was designed in accordance with the declaration of Helsinki. The protocol was approved by and carried out in accordance with the recommendations of the local ethics committee (Maastricht University Medical Center, NL73492.068.20/METC 20–087). Patients provided written informed consent prior to participation and additional travel expenses were reimbursed.

## Results

Nine patients were tested, each on all three vestibular electrodes. This resulted in a total of 27 tested electrodes. The full dataset with VOR results across subjects and electrodes is presented in Supplementary material Table 1. Quantitative video-oculography was not possible on the intraoperative recordings of VCI-8 due to a glare distorting pupil detection.

### Predictive value of intraoperative VOR

An overview of the intra- and postoperative VOR presence is provided in Table 2. The McNemar’s test implied no difference between intra- and postoperative VOR presence (*χ*^*2*^ *= 2.25*,* df = 1*,* p-value = 0.13*). The positive predictive value for the intraoperative VOR in predicting the presence of a postoperative VOR was 1. The negative predictive value of the intraoperative VOR for predicting the absence of a postoperative VOR was 0.86.

**Table 2:** Overview of the number of electrodes with and without intra- and postoperative vestibulo-ocular reflex (VOR) presence in nine patients with a vestibulo-cochlear implant

		Presence of postoperative VOR	
		Yes	No
Presence of intraoperative VOR	Yes	20	0
	No	4	3

### Activation threshold

**Fig. 1:**
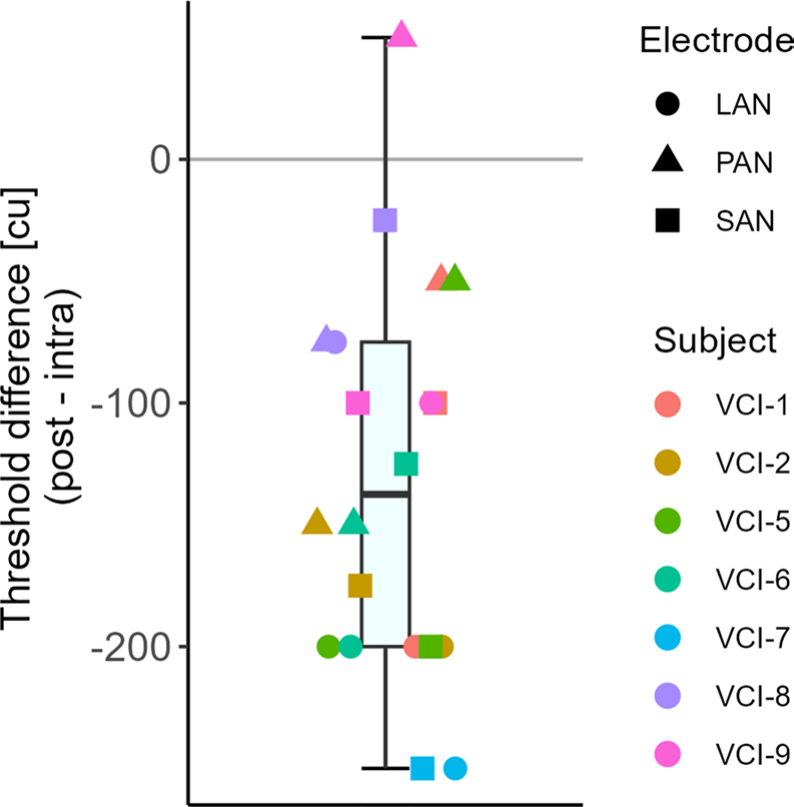
Difference between intra- and postoperative VOR activation thresholds. Only electrodes with intra- and postoperative VOR presence were included. Boxplot represents median and quartiles. LAN = lateral ampullary nerve; SAN = superior ampullary nerve; PAN = posterior ampullary nerve; cu = current unit (1 cu ~ 1 mA)

VOR thresholds were, for all but one case (*n* = 20), lower in the postoperative session compared to the intraoperative measurements (Fig. 1A). The mean difference was 131.25 cu (postoperative lower; SD 80 cu, ranging from − 250 to 50 cu). The paired two-sided t-test showed a significant difference between intra- and postoperative thresholds (*p* < 0.001, *t=−4.54*,* df = 13*).

### VOR velocity

**Fig. 2:**
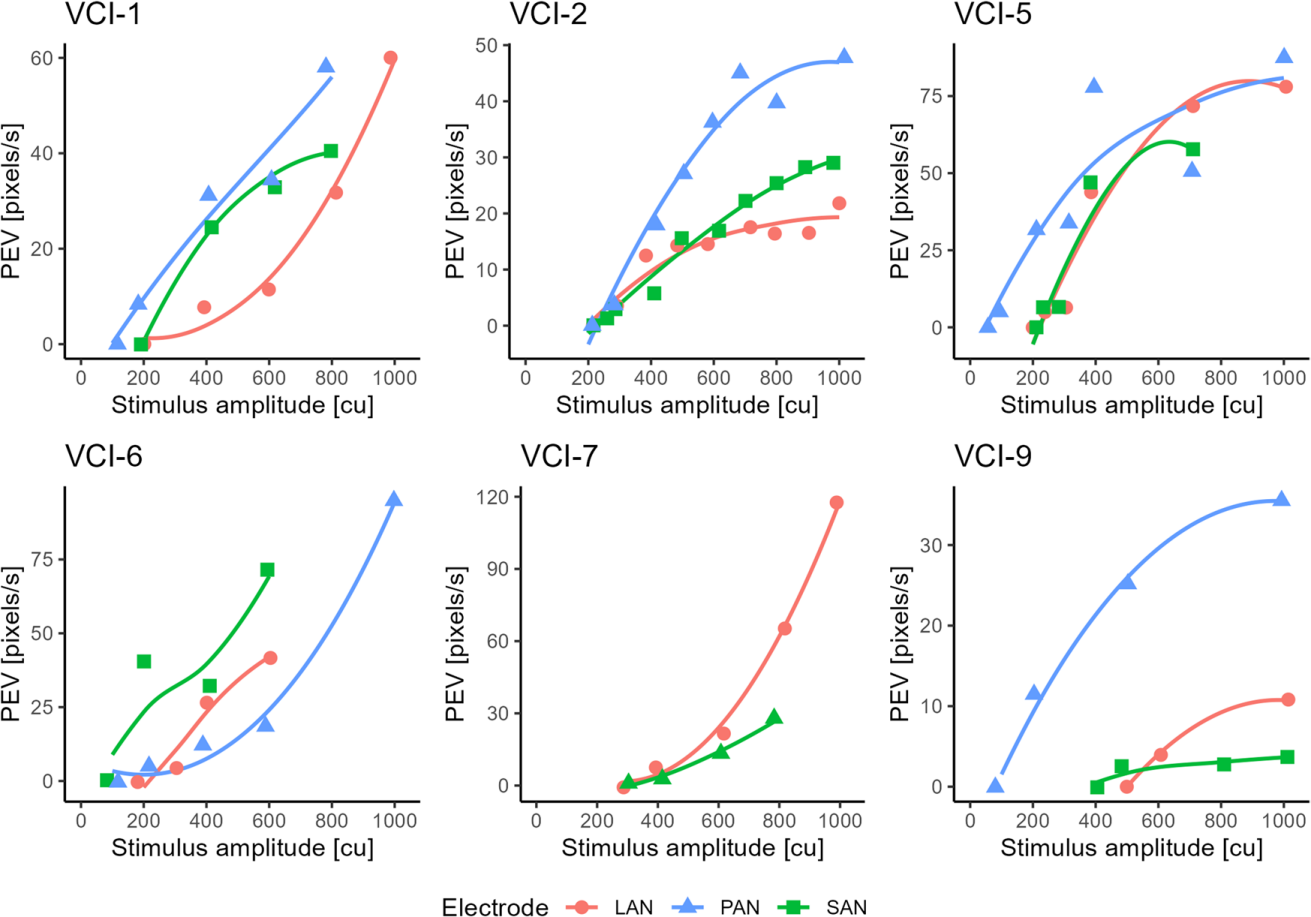
Peak eye velocity of intraoperative eye responses as a function of different stimulation levels, per subject. Markers were connected using a spline fit. Only electrodes with an intraoperative VOR are visualized. VCI-8 was excluded since quantitative video-oculography was not possible due to a glare distorting pupil detection. LAN = lateral ampullary nerve; SAN = superior ampullary nerve; PAN = posterior ampullary nerve; cu = current unit (1 cu ~ 1 mA)

In all electrodes with an intraoperative VOR (*n* = 20), the intraoperative peak eye velocity (pixels/s) was positively, non-linearly, related to the stimulation amplitude (Fig. 2). In all cases with an intraoperative VOR, the postoperative VOR eye velocity was higher (in pixels/s). The mean difference was 46.75 pixels/s (postoperative higher; SD 35.0 pixels/s, ranging from 4.75 to 111.75 pixels/s). Due to variations in the position of the goggles, the velocity in pixels per second could not be strictly quantitatively compared. However, the comparison between pixels per second and calibrated degrees per second showed a high correlation between both measures (R^2^ = 0.82, supplementary material Fig. 1). This implied that the observed difference between intra- and postoperative sessions most likely represented an actual difference in eye velocity. Based on the regression shown in Supplementary material Fig. 1, the average difference of 46.75 pixels per second was estimated to represent a difference of 78 degrees per second.

### VOR alignment

**Fig. 3:**
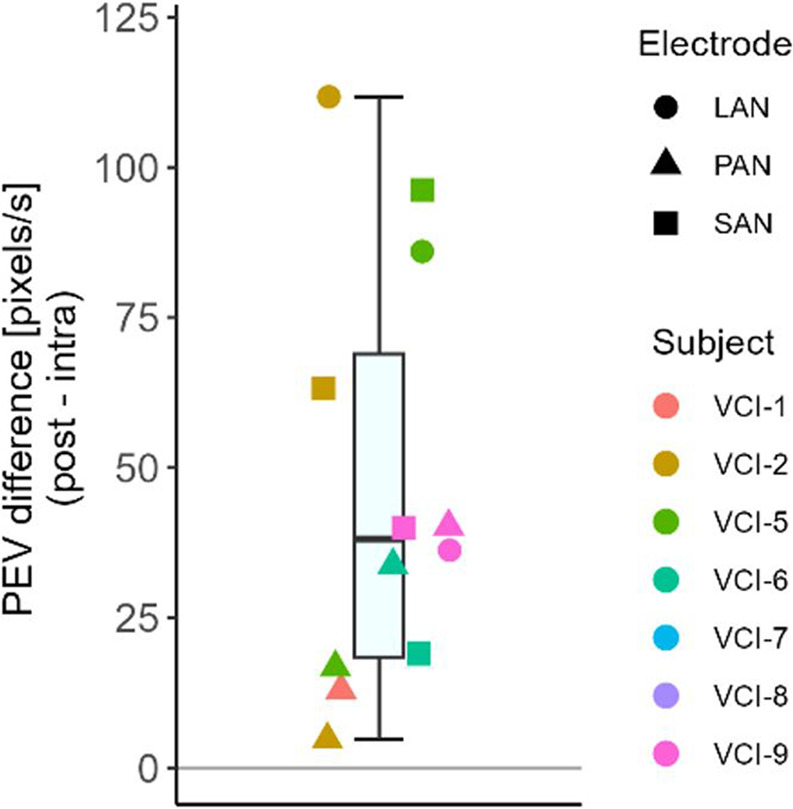
Peak eye velocity difference between intra- and postoperative measurements. The reader should keep in mind that due to variations in position of the goggles, the velocity in pixels per second cannot be strictly quantitatively compared between sessions. VCI-8 was excluded since quantitative video-oculography was not possible due to a glare distorting pupil detection. LAN = lateral ampullary nerve; SAN = superior ampullary nerve; PAN = posterior ampullary nerve

The average misalignment difference between the intra- and postoperative measurements at amplitude threshold plus 100 cu was 6 degrees (Fig. 3A, intraoperative higher, SD 20 degrees, ranging from − 37 to 22 degrees). The paired two-sided t-test showed no significant difference between intra- and postoperative misalignment at threshold plus 100 cu (*p* > 0.05,* t=−1.20*,* df = 14*).

**Fig. 4:**
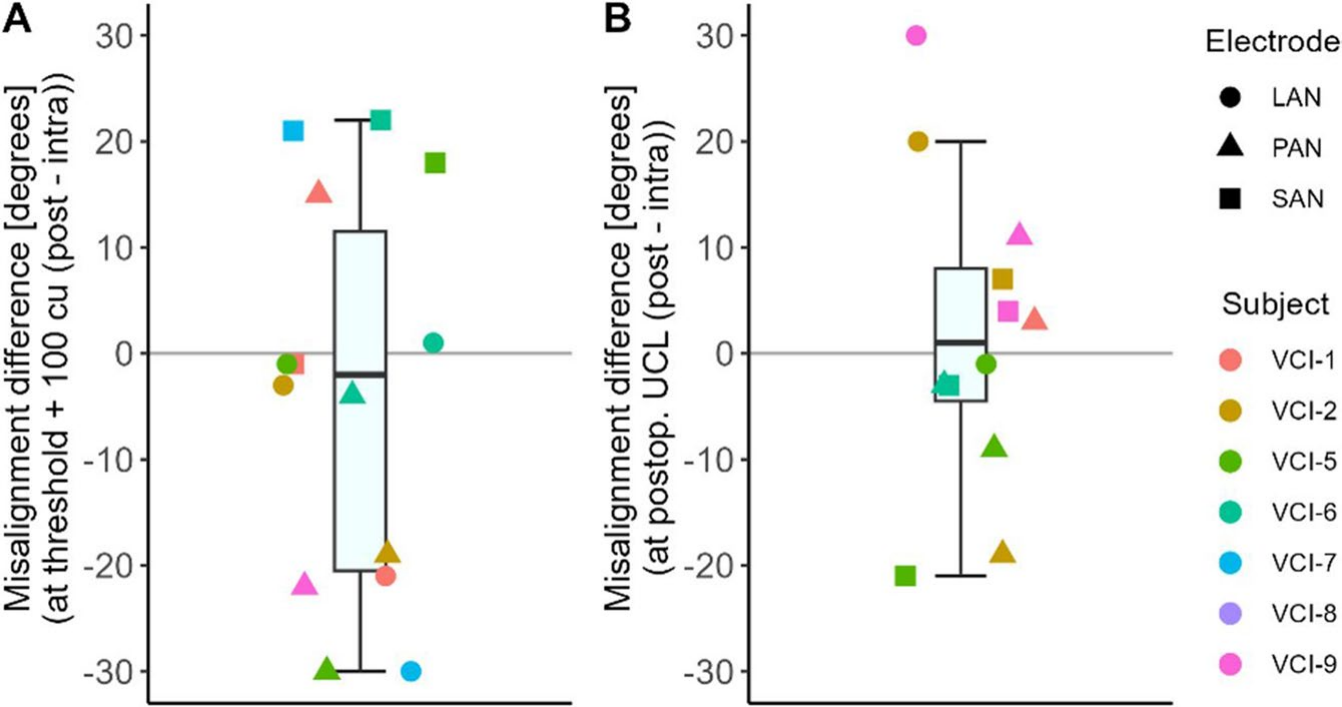
Comparison between intra- and postoperative VOR misalignment. Only electrodes with intra- and postoperative VOR are included. VCI-8 was excluded since quantitative video-oculography was not possible due to glare distorting pupil detection. **(A)** Measurements at the postoperative upper comfortable limit (UCL); **(B)** Measurements with stimulation at threshold plus 100 cu. LAN = lateral ampullary nerve; SAN = superior ampullary nerve; PAN = posterior ampullary nerve. VCI-8 was excluded since quantitative video-oculography was not possible due to a glare distorting pupil detection

The average misalignment difference between the intra- and postoperative measurements at the postoperative upper comfortable limits was 1.58 degrees (Fig. 4B, postoperative higher, SD 15 degrees, ranging from − 21 to 30 degrees). The paired two-sided t-test showed no significant difference between intra- and postoperative misalignment at the postoperative upper comfortable limit (*p* > 0.05,* t=−0.37*,* df = 11*) (Fig. 4).

## Discussion

This study aimed to evaluate the predictive value of intraoperative VOR measurements for the postoperative functionality of the vestibular implant. This is a first step to explore the usability of the VOR as an intraoperative indicator of electrode position. It was found that the intraoperative VOR has a high positive predictive value (1.0) for the presence of a postoperative VOR. Additionally, the intraoperative VOR was found to have a high, though not perfect, negative predictive value (0.86) for the absence of a postoperative VOR. The VOR activation threshold and VOR eye velocity were lower in the postoperative sessions compared to the intraoperative measurements. The intraoperative VOR eye velocity was positively related to the stimulation amplitude. Furthermore, intraoperative VOR alignment was not significantly different from the postoperative VOR alignment.

The presence of an intraoperative VOR was highly predictive for the presence of a postoperative VOR (positive predictive value of 1). However, an absent VOR intraoperatively, did not always imply an absent VOR postoperatively (postoperative predictive value of 0.86). This latter might challenge the use of the VOR an indicator of electrode position. After all, when the VOR is absent intraoperatively, it is unknown whether the potential absence is caused by suboptimal electrode positioning or by other factors. Other factors could be the suppression of the VOR by the general anesthesia, the lack of excitable neural substrate, or the underlying etiology of vestibulopathy [9, 13, 14, 16, 17]. Moreover, the vestibular implant may still provide benefits by delivering vestibular information, even in the absence of a VOR response. Therefore, electrode positioning remains important. Altogether, these findings imply that the VOR cannot be used as the sole means to evaluate electrode position.

In four electrodes (subjects VCI-4 (all electrodes) and VCI-7 (PAN electrode)) a VOR response was found postoperatively, but not intraoperatively. Most likely, the suppressive effect of the general anesthesia caused the differences between the intra- and postoperative responses. Interestingly, the postoperative peak eye velocities of all electrodes of VCI-4 were very low (< 4 °/s). It can be hypothesized that these small responses could be diminished by anesthesia. The postoperative VOR maximum eye velocity of VCI-7 PAN was higher: approximately 50 °/s. However, it was still lower compared to the other VCI-7 electrodes (100 °/s and 170 °/s for LAN and SAN). Conversely, other factors do not explain the difference between the intra- and postoperative VOR in these four electrodes. For example, all four electrodes had a postoperative activation threshold of ≤ 250 cu. This was well below the intraoperative maximum stimulation amplitude of 1000 cu. Moreover, electrode positions were equal when performing intra- and postoperative testing [18]. Lastly, postoperative testing was performed within weeks after surgery, which would make a neural change unlikely.

The hypothesis of anesthesia causing the difference between intra- and postoperative VOR responses, is further supported by the fact that all electrodes showed a lower threshold of stimulation postoperatively compared to intraoperative measurements. Although the threshold is probably affected by the general anesthesia, it might be considered as a dynamic intraoperative marker for electrode positioning. A lower threshold, as a result of manipulating the electrode, still suggests a better position of the electrode. However, more research is needed to determine the correlation between the threshold and electrode position.

The findings of this study suggest that the eye velocity is also a potential marker for the effectiveness of the stimulation. The intraoperative measurements showed that the eye velocity of the VOR increased with increased stimulation amplitudes. The same phenomenon was observed previously in post-operative measurements [9]. Postulating that a better electrode positioning results in a higher efficacy of stimulation, gives rise to the possibility to use the eye velocity as a marker for electrode position. The electrode could be stimulated at a supra-threshold level while manipulating the electrode, during which the eye velocity could be monitored. An increase in eye velocity would indicate a more optimal electrode position, while a decrease would indicate a less optimal electrode position.

Next to the eye velocity of the VOR response, the alignment was a factor of interest. A prerequisite for intraoperative use is that the intraoperative alignment of the response accurately predicts the postoperative alignment. The results in this study indicated no significant overall difference in misalignment between intra- and postoperative sessions. However, at the individual electrode level, the misalignment could differ up to 30 degrees (both higher and lower). This suggests that, based on the tested electrodes, the misalignment should be interpreted with caution when used as a marker for electrode position. The question remains what caused the difference in alignment. The general anesthesia appeared to suppress the VOR, as indicated by the elevated thresholds and lower eye velocity. However, the adjacent nerve ends would be equally affected by the anesthesia. Therefore, it could be hypothesized that alignment would not be significantly influenced by anesthesia. A measurement artefact could play a role, since misalignment measurements show a degree of variability [9]. Furthermore, different goggle positions between sessions could have added to this variability.

### Implementation of VOR testing during surgery

The findings of this study can be combined to optimize electrode positioning during surgery. In case no VOR response is found, it would be worth changing the position of the electrode until a response appears. In case a VOR response is found, electrode positioning could be optimized by looking for the maximum eye velocity. In other words: multiple stimulations could be applied when positioning the electrodes. Monitoring the eye velocity while changing electrode position, could provide information about the position of the electrode relative to excitable neurons. A higher eye velocity would indicate a better electrode position. An alternative would be to look for the lowest threshold. However, determining thresholds at each electrode position, would be more time-consuming.

In the future, vestibular implant surgery might be possible under local anesthesia, instead of general anesthesia [19]. That could resolve the effect of general anesthesia on the VOR. Additionally, local anesthesia allows intraoperative patient cooperation, enabling calibration of the VideoFrenzel goggles [20, 21]. This would make it possible to perform intraoperative quantitative eye tracking.

### Limitations

At this stage of vestibular implant research, sample size remains an inherent limitation. This study, which included nine subjects, represents a relatively large sample for the field. However, the small sample size presents a challenge for statistical analysis in drawing conclusive results. Furthermore, potential factors of influence, such as inter-subject variability and type of nerves being stimulated (LAN/SAN/PAN), could not be fully elucidated. Lastly, the lack of intraoperative video-oculography goggles calibration limited the quantitative comparison between intra- and postoperative measurements.

## Conclusion

Intraoperative VOR responses during vestibular implant surgery differ from postoperative responses, likely due to general anaesthesia. Despite this discrepancy, the presence of an intraoperative VOR is a strong predictor of postoperative VOR presence. Furthermore, intraoperative VOR eye velocity increases with higher stimulation amplitudes. These findings suggest that intraoperative VOR testing may aid in optimizing electrode placement during vestibular implant surgery.

## Supplementary Information

Below is the link to the electronic supplementary material.

Supplementary Material 1
