# Supplementary material for: Exploring the role of the electrically evoked Vestibulo-Ocular reflex in vestibular implant surgery
Source: Eur Arch Otorhinolaryngol. 2025 Aug 13;283(1):131–9. doi: 10.1007/s00405-025-09635-y (PMC12904894; doi:10.1007/s00405-025-09635-y)
Supplement: Supplementary file 1 — Supplementary Material 1 [file 405_2025_9635_MOESM1_ESM.docx]

# Supplementary material

*Table 1: intra- and postoperative response characteristics for all subject and electrodes. LAN-electrode targets the lateral ampullary nerve, SAN the superior ampullary nerve and PAN the posterior ampullary nerve. Alignment describes the direction of the slow phase of the eye response as a result of stimulation, where 0 degrees is left, 90 upwards, 180 right, 270 down (from subjects perspective). VOR = vestibulo-ocular reflex; PEV = peak eye velocity; NR = no response; NA = not available; UCL = upper comfortable limit; cu = current unit.*

| Subject | Electrode | VOR  response present | |  | VOR activation threshold  (cu) | |  | PEV at postoperative UCL  (pixels/s) | |  | PEV at postoperative UCL  (°/s) |  |  | Misalignment at threshold + 100 cu (degrees) | |  | Misalignment at postoperative UCL  (degrees) | |  | Postoperative UCL  (cu) |
| --- | --- | --- | --- | --- | --- | --- | --- | --- | --- | --- | --- | --- | --- | --- | --- | --- | --- | --- | --- | --- |
|  |  | intra | post |  | intra | post |  | intra | post |  | post |  |  | intra | post |  | intra | post |  | post |
| VCI-1 | LAN | yes | yes |  | 300 | 100 |  | NR | 21 |  | 41 |  |  | 36 | 15 |  | NR | 26 |  | 300 |
|  | SAN | yes | yes |  | 250 | 150 |  | NR | 6.5 |  | 24 |  |  | 14 | 13 |  | NR | 96 |  | 200 |
|  | PAN | yes | yes |  | 200 | 150 |  | 8.5 | 21.5 |  | 10 |  |  | 1 | 16 |  | 13 | 16 |  | 200 |
|  |  |  |  |  |  |  |  |  |  |  |  |  |  |  |  |  |  |  |  |  |
| VCI-2 | LAN | yes | yes |  | 300 | 100 |  | 3.5 | 115.3 |  | 157 |  |  | 9 | 6 |  | 3 | 23 |  | 275 |
|  | SAN | yes | yes |  | 250 | 75 |  | 3 | 66.3 |  | 105 |  |  | 50 | 13 |  | 21 | 28 |  | 300 |
|  | PAN | yes | yes |  | 300 | 150 |  | 18 | 22.8 |  | 30 |  |  | 21 | 2 |  | 21 | 2 |  | 350 |
|  |  |  |  |  |  |  |  |  |  |  |  |  |  |  |  |  |  |  |  |  |
| VCI-3 | LAN | no | no |  | NR | NR |  | NR | NR |  | NR |  |  | NR | NR |  | NR | NR |  | 350 |
|  | SAN | no | no |  | NR | NR |  | NR | NR |  | NR |  |  | NR | NR |  | NR | NR |  | 350 |
|  | PAN | no | no |  | NR | NR |  | NR | NR |  | NR |  |  | NR | NR |  | NR | NR |  | 375 |
|  |  |  |  |  |  |  |  |  |  |  |  |  |  |  |  |  |  |  |  |  |
| VCI-4 | LAN | no | yes |  | NR | 250 |  | NR | 6.8 |  | 3 |  |  | NR | 0 |  | NR | 0 |  | 225 |
|  | SAN | no | yes |  | NR | 200 |  | NR | 7 |  | 2 |  |  | NR | 0 |  | NR | 0 |  | 250 |
|  | PAN | no | yes |  | NR | 250 |  | NR | 5.5 |  | 3 |  |  | NR | 0 |  | NR | 0 |  | 300 |
|  |  |  |  |  |  |  |  |  |  |  |  |  |  |  |  |  |  |  |  |  |
| VCI-5 | LAN | yes | yes |  | 250 | 50 |  | 5 | 91 |  | 187 |  |  | 6 | 5 |  | 9 | 8 |  | 250 |
|  | SAN | yes | yes |  | 250 | 50 |  | 6.5 | 102.8 |  | 220 |  |  | 3 | 21 |  | 22 | 1 |  | 225 |
|  | PAN | yes | yes |  | 100 | 50 |  | 2.8 | 19.5 |  | 80 |  |  | 32 | 2 |  | 22 | 13 |  | 350 |
|  |  |  |  |  |  |  |  |  |  |  |  |  |  |  |  |  |  |  |  |  |
| VCI-6 | LAN | yes | yes |  | 300 | 100 |  | NR | 41 |  | 89 |  |  | 88 | 89 |  | NR | 90 |  | 150 |
|  | SAN | yes | yes |  | 200 | 75 |  | 40.5 | 59.5 |  | 70 |  |  | 8 | 30 |  | 19 | 16 |  | 150 |
|  | PAN | yes | yes |  | 200 | 50 |  | 5.3 | 39 |  | 50 |  |  | 14 | 10 |  | 13 | 10 |  | 150 |
|  |  |  |  |  |  |  |  |  |  |  |  |  |  |  |  |  |  |  |  |  |
| VCI-7 | LAN | yes | yes |  | 400 | 150 |  | NR | 50.5 |  | 85 |  |  | 97 | 67 |  | NR | 65 |  | 300 |
|  | SAN | yes | yes |  | 400 | 150 |  | NR | 39.3 |  | 40 |  |  | 1 | 22 |  | NR | 13 |  | 300 |
|  | PAN | no | yes |  | NR | 200 |  | NR | 20.8 |  | 42 |  |  | NR | 55 |  | NR | 16 |  | 425 |
|  |  |  |  |  |  |  |  |  |  |  |  |  |  |  |  |  |  |  |  |  |
| VCI-8 | LAN | yes | yes |  | 150 | 75 |  | NA | 26.8 |  | 67 |  |  | NA | 1 |  | NA | 38 |  | 525 |
|  | SAN | yes | yes |  | 150 | 125 |  | NA | 19.8 |  | 70 |  |  | NA | 1 |  | NA | 20 |  | 500 |
|  | PAN | yes | yes |  | 150 | 75 |  | NA | 15.8 |  | 50 |  |  | NA | 8 |  | NA | 5 |  | 825 |
|  |  |  |  |  |  |  |  |  |  |  |  |  |  |  |  |  |  |  |  |  |
| VCI-9 | LAN | yes | yes |  | 600 | 500 |  | 4 | 40.8 |  | 80 |  |  | NA | 5 |  | 21 | 51 |  | 550 |
|  | SAN | yes | yes |  | 500 | 400 |  | 2.5 | 42.5 |  | 64 |  |  | NA | 13 |  | 9 | 13 |  | 400 |
|  | PAN | yes | yes |  | 200 | 250 |  | 11.5 | 51.8 |  | 105 |  |  | 37 | 15 |  | 20 | 31 |  | 250 |


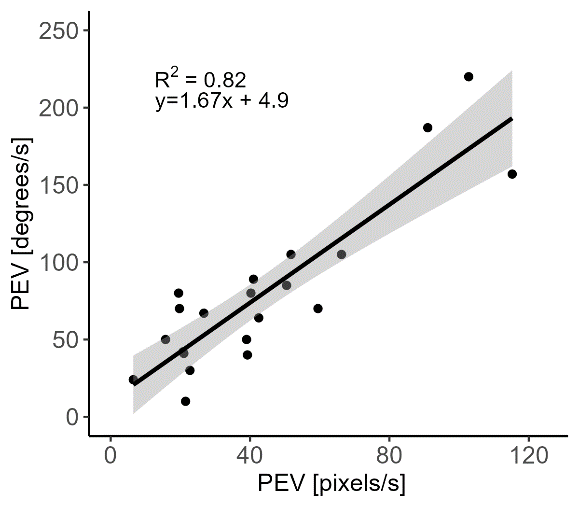


*Figure S1: Peak eye velocity (PEV) comparison measured in pixels per second and degrees per second of the postoperative VOR measurements at upper comfortable limit. Strait line represents linear regression, with confidence interval in gray.*
